# Annotations

**Published:** 1893-07-22

**Authors:** 


### Annotations.

**Cycling and Heart Disease.**

Dr. Edmund Cautley thinks he has come upon a case of heart disease due directly to cycling. The patient is thirty-four years of age. The history is one of five years more or less strenuous exercise on a bicycle. The nature of the affection is the partial rupture of one of the aortic valves. The signs are a slow, heaving, diffused impulse, a prolonged first sound, a diastolic musical murmur over the second left intercostal space, and a slow pulse, fifty-four, somewhat "sudden" in character. There have also been noted some indigestion, and the feeling of pins and needles in the feet. This is a note of warning to which cyclists should give heed. Dr. Cautley does not wish it to be understood that cycling itself is injurious; he only desires that cyclists, old and young, will indulge their pastime intelligently. He is of opinion that the fact of this particular individual having commenced to cycle somewhat late in life, has had something to do with the unwelcome result. A second factor was probably an attack of influenza. But the most important circumstance of all appears to have been that the victim cycled too hard. For the last five years he has "gone in largely"; he has not competed in races, but he has "raced" a great deal "against time," his special efforts being made on Saturdays when he has tried more or less frequently and strenuously to increase the pace between London and Brighton. All this seems to us matter of importance. Cycling is good, but excessive cycling may be very mischievous. We fully anticipate more cases of this and similar injuries during the next few years. Middle-aged and old men especially should cycle with moderation; but all classes of people should avoid continual cycling overstrain as carefully as they would avoid continual overwork or continual loss of sleep. It may be taken as a law which knows no variation that in no way can nature be outraged with impunity.

**Cholera, Tea, and Trade.**

Dr. J. R. Stocker, of the Board of Trade, publishes an article in the *Nautical Magazine* for the special instruction of sailors in the art of avoiding cholera. The article contains much matters of the first importance on the general questions of quarantine, the "watering" of ships at different ports, proposals for medico-consular inspection and reporting at dangerous ports, with other suggestions, which it is quite impossible intelligently to condense into a few lines. But the pith and marrow of Dr. Stocker's earnest pleading with sailors and all who "go down to the sea in ships" lies in the second and last paragraphs of his article. Cholera, says Dr. Stocker, is a "water-borne" disease; therefore beware of the drinking water under all conceivable circumstances. "The Chinese," he goes on to state, "are said to be exempt from cholera because they drink nothing but tea." Is it the tea which assures the exemption, or is it the fact that proper tea can only be made with boiling water? If the Chinese are to a large extent exempt from cholera we may be quite sure that it is because they never, on any consideration, drink water which has not previously been boiled. These are the points of Dr. Stocker's contention: Take out in ships nothing but pure water; take in water at no port on the voyage, unless you are assured on medical as well as consular authority, that the water is fairly pure; but in order to make assurance doubly sure, see that all the water used for drinking purposes is filtered and boiled, but especially boiled. By way of showing the British nation a practical example, Dr. Stocker tells us what America does to preserve her people from cholera. She is not content with quarantine, or with medical inspection of ships and crews on their arrival in port. She is far more thorough. America insists upon a clean bill of health at the port of departure, and not a "consular" bill only, but a "medical." This is going to the root of the matter. There is no doubt that if ships start out on their voyages with a clean bill of health; if they take only pure water for the voyage; and if they filter and boil all the water they use, there will be practically no carrying of cholera from country to country by trading vessels, and no interruption of trade.

**The Ups and Downs of Doctors.**

The least selfish of human beings cannot avoid taking an interest in his own personal affairs, and doctors may be excused if they sometimes take financial stock of their own profession. Are medical men as well off now as they were fifty years ago, or are they poorer as a class? That they are better educated and of much more value to the public we are quite certain. But does the public value them more highly? Does it pay them better? These are questions which we cannot answer in the affirmative. A little more than half a century ago—or, to be quite precise, in the year 1835—the British Medical Benevolent Fund was founded, and its object was to help poor medical men, their widows and their orphans, when reduced to the very narrowest of distressful straits. The help contemplated to be given was very small, because the resources of the fund were small. But in looking over the list of beneficiaries year by year for the fifty-eight years of the fund's existence, we are struck by the extreme smallness of the numbers requiring (or, at any rate, receiving) relief in the early years of the fund's life compared with the many hundreds of those who have applied for and received it during the later decades. Dividing the history of the fund into septennial periods, we may compare the first septennium with the last. If we take the period from 1835 to 1842, we find that in the first year, according to the annual report just issued, there were no claimants at all for the benefits of the fund; in the second year there were 2; in the third, 5; in the fourth, 5; in the fifth, 4; in the sixth, none; and in the seventh, none; or a total of 16 for the septennium. Now let us take the last septennium, from the year 1885 to 1892. In the first of these years 183 persons received grants; in the second, 166; in the third, 160; in the fourth, 159; in the fifth, 159; in the sixth, 159; and in the seventh, 140; or a total of 1,126, as against 16 in the first septennium. These figures are, at any rate, striking. They show, of course, the conspicuous success and usefulness of the fund. It is to be feared that they show also a gradual deterioration in the financial position of the rank and file of the profession. There are many other circumstances which point to the same conclusion, and we cannot but feel that the time has fully arrived for the profession as a whole to take stock of its progress, and to initiate a determined effort to make life worth living, even for the hard-worked "night and day" doctor.

